# Retrieving the *in vivo* Scopoletin Fluorescence Excitation Band Allows the Non-invasive Investigation of the Plant–Pathogen Early Events in Tobacco Leaves

**DOI:** 10.3389/fmicb.2022.889878

**Published:** 2022-04-29

**Authors:** Giovanni Agati, Cecilia Brunetti, Lorenza Tuccio, Ilaria Degano, Stefania Tegli

**Affiliations:** ^1^Istituto di Fisica Applicata “Nello Carrara” (IFAC), Consiglio Nazionale delle Ricerche, Sesto Fiorentino, Italy; ^2^Consortium INSTM-Italian Interuniversity Consortium for Science and Technology of Materials, Firenze, Italy; ^3^Istituto per la Protezione Sostenibile delle Piante (IPSP), Consiglio Nazionale delle Ricerche, Sesto Fiorentino, Italy; ^4^Dipartimento di Chimica e Chimica Industriale, Università di Pisa, Pisa, Italy; ^5^Department of Agriculture, Food, Environment and Forestry (DAGRI), University of Florence, Sesto Fiorentino, Italy

**Keywords:** fluorescence spectroscopy, non-destructive detection, scopolin, scopoletin, *Nicotiana tabacum*, *Pseudomonas syringae*, hypersensitive response, type III secretion system

## Abstract

In this study, we developed and applied a new spectroscopic fluorescence method for the *in vivo* detection of the early events in the interaction between tobacco (*Nicotiana tabacum* L.) plants and pathogenic bacteria. The leaf disks were infiltrated with a bacterial suspension in sterile physiological solution (SPS), or with SPS alone as control. The virulent *Pseudomonas syringae* pv. *tabaci* strain ATCC 11528, its non-pathogenic *ΔhrpA* mutant, and the avirulent *P. syringae* pv. *tomato* strain DC3000 were used. At different post-infiltration time–points, the *in vivo* fluorescence spectra on leaf disks were acquired by a fiber bundle-spectrofluorimeter. The excitation spectra of the leaf blue emission at 460 nm, which is mainly due to the accumulation of coumarins following a bacterial infiltration, were processed by using a two-bands Gaussian fitting that enabled us to isolate the scopoletin (SCT) contribution. The pH-dependent fluorescence of SCT and scopolin (SCL), as determined by *in vitro* data and their intracellular localization, as determined by confocal microscopy, suggested the use of the longer wavelength excitation band at 385 nm of 460 nm emission (F_385_460_) to follow the metabolic evolution of SCT during the plant–bacteria interaction. It was found to be directly correlated (*R*^2^ = 0.84) to the leaf SCT content, but not to that of SCL, determined by HPLC analysis. The technique applied to the time-course monitoring of the bacteria–plant interaction clearly showed that the amount and the timing of SCT accumulation, estimated by F_385_460_, was correlated with the resistance to the pathogen. As expected, this host defense response was delayed after *P. syringae* pv. *tabaci* ATCC 11528 infiltration, in comparison to *P. syringae* pv. *tomato* DC3000. Furthermore, no significant increase of F_385_460_ (SCT) was observed when using the non-pathogenic *ΔhrpA* mutant of *P. syringae* pv. *tabaci* ATCC 11528, which lacks a functional Type Three Secretion System (TTSS). Our study showed the reliability of the developed fluorimetric method for a rapid and non-invasive monitoring of bacteria-induced first events related to the metabolite-based defense response in tobacco leaves. This technique could allow a fast selection of pathogen-resistant cultivars, as well as the on-site early diagnosis of tobacco plant diseases by using suitable fluorescence sensors.

## Introduction

During the interaction with their potential host plants, the pathogenic microorganisms are exposed to a wide array of antimicrobial compounds. Phytoalexins are among the most important plant secondary metabolites, playing a key role in the host immune response. These low molecular weight compounds are newly produced and locally accumulate in the plant tissues following the application of biotic or abiotic stresses (Ahmed and Kovinich, 2021). Despite their common and conserved biological activity, phytoalexins belong to several chemical families, which can be sometimes specifically associated to different botanical families.

The phytoalexin scopoletin (6-methoxy-7-hydroxycoumarin) (SCT) is synthesized through the phenylpropanoid pathway by many plant species. The SCT and other coumarins are beneficial for plants because of their antimicrobial and antioxidant properties (Stringlis et al., 2019), as well as for their role in iron uptake (Robe et al., 2020). In addition, coumarins are rather valuable for humans because of their multiple pharmacological properties (Stefanachi et al., 2018).

Together with capsidiol, SCT represents the major phytoalexin in tobacco (*Nicotiana tabacum* L.), and its accumulation in the leaf tissues has been correlated with *N. tabacum* resistance toward several phytopathogens (Ahuja et al., 2012). Actually, unless a stress is applied, SCT is not generally present in the plant tissues in its free state, because for the most part it accumulates in the vacuoles as its 7-β-D-glucoside form, named scopolin (SCL) (Hino et al., 1982).

Similar to most coumarins, SCT and SCL are strongly fluorescent under UVA excitation, as already reported in the 1960s (Crosby and Berthold, 1962; Dieterman et al., 1964), and thus their presence in plant tissues can be detected by fluorescence spectroscopy. An *in vivo* bright blue autofluorescence under UV excitation was first observed in tobacco leaf tissues treated with a hypersensitive reaction-inducing elicitor, and it was related to the SCT accumulation into plant tissues upon elicitor exposure (Dorey et al., 1997).

A correlation between the presence of SCL and SCT and the UV-excited blue fluorescence was then confirmed in tobacco leaves infected with the tobacco mosaic virus (TMV) (Chong et al., 2002; Costet et al., 2002).

In addition, SCT was linked to the blue autofluorescence detected 4 days after the infection by *B. cinerea* of the tobacco resistant Petit Havana cultivar (El Oirdi et al., 2010).

The resistance of *N. attenuata* to *A. alternata* was also associated with a strong blue autofluorescence, depending on a functional feruloyl-CoA 6′-hydroxylase 1, that is the essential enzyme in the SCT biosynthesis. Accordingly, the accumulation of SCT and SCL, the fluorescence intensity, and the resistance to *A. alternata* infection were higher in young leaves than in fully expanded leaves (Sun et al., 2014).

Overall, these evidences suggest that the non-destructive optical sensing of *in planta* fluorescence could be employed for the quantitative monitoring of the very early defense events related to the attack by a virulent or avirulent pathogen. So far, the multispectral fluorescence and thermal imaging have been successfully proved to detect the first steps of the interaction occurring between plants and their viral, bacterial and even fungal pathogens (Chaerle et al., 2007; Barón et al., 2016; Hupp et al., 2019). A similar non-destructive approach may represent an efficient and cost-effective strategy for monitoring the plant health status in the field. Time-course analyses on the very same plant would also be possible, avoiding most of the variability inherent to the biological samples. Moreover, its application on a laboratory scale would be a valid alternative to the current methods used for the quantitative evaluation of coumarins in leaf exudates (El Modafar et al., 1995; Churngchow and Rattarasarn, 2001) or leaf extracts (Sun et al., 2014). However, the fluorescence-based techniques used up to now are based on a single excitation wavelength and the related blue emission band detection, usually calibrated against SCT, and they cannot distinguish between the contribution to fluorescence of the coumarins present in the tobacco plants.

The present work aimed to characterize the *in vivo* fluorescence signals of *N. tabacum* bacteria-infected leaves by spectroscopic methods. Here, for the first time, the *in vivo* blue fluorescence contributions given by SCL and SCT, were discriminated by the elaboration of the fluorescence excitation spectra from tobacco leaves infiltrated with virulent, avirulent and even non-pathogenic *Pseudomonas syringae* strains. This allowed the validation of the spectroscopic approach as a new and efficient non-destructive technique for diagnostic monitoring of the very early events of the plant–pathogen interaction.

## Materials and Methods

### Plants and Bacteria Used

The tobacco plants (*N. tabacum* var. ITB 6178) (Bergerac Seed & Breeding Soc., Bergerac, France) were grown into pots containing a compost–soil substrate, under controlled conditions (16 h day/8 h night, at temperature 22°C/18°C, and 70% relative humidity). Fully expanded leaves from 10 to 14 weeks old plants were used for the experiments.

*P. syringae* pv. *tomato* DC3000, *P. syringae* pv. *tabaci* ATCC 11528 and its *ΔhrpA* mutant, constructed according to Cerboneschi et al. (2016) (Tegli, unpublished data), were routinely grown at 26°C in liquid or agarised King’s B medium (KB) (King et al., 1954), and long-term stored in 40% (v/v) aqueous glycerol solution at −80°C.

### Leaf Disks Bacterial Infiltration

Overnight liquid bacterial cultures (20 ml KB) were incubated at 26°C, under continuous orbital shaking (100 rpm). Then, the bacterial cultures were centrifuged at 8.000*g* for 10 min, and the pellet washed twice in sterile physiological solution (SPS) (0.85% NaCl in distilled water), and resuspended up in SPS to a final OD_600_ = 0.5 (approximately 0.5 × 10^8^ CFU ml^–1^).

Leaf disks were obtained from the youngest fully expanded leaves of adult plants, by using a cork borer (11-mm diameter). From each freshly detached leaf, 35 disks were obtained and placed into Petri dishes containing SPS until used for bacterial infiltration. For each bacterial treatment, a sample of 10 disks randomly selected was used, and three replicates were made for each inoculation trial. Each leaf disk sample was then placed in a 50-ml sterile centrifuge tube containing a 20-ml bacterial suspension, and then infiltrated under vacuum, according to Johansson with minor modifications (Johansson et al., 2015). As a negative control, SPS was used for infiltration. For each treatment, infiltrated leaf disks were separately processed. After the vacuum infiltration step, the leaf disks were rinsed 3 times with sterile distilled water, then transferred on sterile filter paper to dry the excess of water, before to be incubated into sterile empty Petri dishes (90-mm diameter) in moist sterile conditions at 28°C in the dark.

### *In situ* and *in vitro* Fluorescence Spectroscopy

Scopolin (PhytoLab GmbH & Co. KG, Vestenbergsgreuth, Germany), SCT, and chlorogenic acid (CGA) (both from Sigma–Aldrich, Milano, Italy) were dissolved in ethanol (0.3–0.6 mg ml^–1^) and then diluted with a phosphate buffer solution at different pH values (2.6, 5.4, 6.7, 7.2, 7.6, and 8.5), to achieve final concentrations ranging between 30 and 70 μM. The actual pH of solutions was measured with a HI 2211 pH meter (Hanna Instruments, Woonsocket, RI, United States). All chemicals and solvents used were of HPLC grade.

The UV-absorbance spectra were recorded by a Jasco V-560 spectrophotometer (Jasco, Tokyo, Japan). The fluorescence spectra were recorded by a Cary Eclipse spectrofluorimeter (Agilent Technologies, Cernusco sul Naviglio, Milano, Italy) in a 10 × 10-mm quartz cuvette, after diluting the sample solutions to get an absorbance of about 0.1 at the excitation wavelength. Setting of acquisition parameters, gain and slit width, were adjusted for each sample to avoid signal saturation.

To compare the *in vitro* fluorescence efficiency of compounds, the same acquisition parameters (exc/em slits = 2.5/2.5 nm, PMT voltage = 850 V) were used and fluorescence intensities were divided by the absorbed fraction (1 - 10^–^^Aλ^^exc^) at the excitation wavelength.

The *in vivo* fluorescence spectra on leaf disks were acquired at different times of incubation by the above described spectrofluorimeter equipped with a bifurcate fiber bundle on a spot of 6-mm diameter, with a PMT voltage of 850 V, slit widths of exc/em monochromators of 5/10 nm and 10/5 nm for the excitation and emission acquisition, respectively. The scan rate was 600 nm min^–1^ and the averaging time was 0.2 s.

The excitation spectra were corrected for the wavelength dependency of the detector system.

### Confocal Laser Scanning Microscopy

Leaf disks (11-mm diameter) were mounted in water on microscope slides and observed by using a Leica TCS SP8 confocal upright microscope (Leica Microsystems CMS, Mannheim, Germany) equipped with a 63× objective (HC PL APO CS2 63 × /1.40 OIL).

The laser excitation at 405 nm was set at 40% of maximal intensity and used to acquire blue fluorescence of coumarins over the 430–498-nm emission spectral band, with gain at 850. The chlorophyll fluorescence was acquired under 638-nm excitation at 35% of maximal intensity over the 695–765-nm emission spectral band, with gain at 632. The image spatial calibration was between 0.057 and 0.241 μm pixels^–1^.

### Extraction and Analysis of Phenolic Compounds

Leaf disks (30 mg/sample) were ground with liquid nitrogen and extracted 3 times with 0.5 ml of 75% MeOH/H_2_O adjusted to pH 2.5 with formic acid. The supernatant was defatted with 1 ml of *n*-hexane and then centrifuged before 20 μl of the extract was injected into the Jasco HPLC system. It consisted of a Jasco PU-4180 RHPLC pump, a MD-4010 PDA detector followed in series by a FP-4025 fluorescence detector, an AS-4050 autosampler injection valve and LC-Net II/ADC interface [Jasco Europe S.R.L., Cremella (LC), Italy].

The LC separation was performed on a reverse phase column (Kinetex Phenomenex C18, 150 × 4.6 mm, 5 μm) maintained at 25°C (Jasco CO-4060 column oven) and using mobile phase A (water 0.1% formic acid) and mobile phase B (CH_3_CN 0.1% formic acid) in a gradient program with a flow of 0.6 ml min^–1^: 0-2 min 100% A, 2-22 min 100-80% A, 22-24 min 80% A, 24-34 min 80-10% A, 34-44 min 10-0% A, 44-50 min 100% B. Then SCT, SCL, and CGA were identified by comparison of their retention times and UV spectral characteristics with those of authentic standards and quantified using five-point calibration curves of authentic standards. Chlorogenic acid was quantified by absorbance at 330 nm, whereas SCT and SCL were quantified using fluorescence chromatograms with excitation wavelength at 340 nm and emission responses at 460 nm, with the PMT gain value set at 100.

A further HPLC-ESI-MS/MS analysis was used to investigate the leaf extract composition and the possible assignment of the minor fluorescent compounds with retention times longer than 30 min.

For the HPLC-MS analysis the system consisted of an HPLC 1200 Infinity, coupled with a quadrupole-time of flight mass spectrometer Infinity Q-ToF 6530 detector through a Jet Stream ESI interface (Agilent Technologies). The ESI conditions were as listed as follows: Drying and sheath gas N_2_, purity > 98%, temperature 350°C, flow 10 L/min and temperature 375°C, flow 11 L/min, respectively; capillary voltage 4.5 kV; nebulizer gas pressure 35 psi. The fragmentor, nozzle, skimmer, and octapole RF voltages were set at 175, 1000, 65, and 750 V, respectively. The high-resolution MS and MS/MS acquisition range was set from 100 to 1000 m/z in in positive mode, with acquisition rate 1.04 spectra/s. For the MS/MS experiments, 30 V were used (collision gas N_2_).

The eluents used for the HPLC–ESI–Q–ToF analyses were LC–MS grade (Sigma–Aldrich, United States) water (eluent A) and acetonitrile (eluent B), both added with 0.1% v/v formic acid (98% purity, J.T. Baker, United States).

The chromatographic separation was performed at 30°C on an analytical reversed-phase column Poroshell 120 EC-C18 (3.0 × 75-mm, 2.7 μm) with a Zorbax pre-column (4.6 × 12.5 mm, 5 μm), both Agilent Technologies. The flow rate was 0.4 ml/min and the program was 0–2.6 min 5% B, 2.6–15.6 min 5–50% B, 15.6–20.8 min 50–70% B, 20.8–27 min 70–100% B, then held for 7 min. The re-equilibration took 10 min. The injection volume was 20 μl.

### Data Analysis

Statistical analysis and curve fitting were carried out with the SigmaPlot for Windows Version 14.0 software (Systat Software, Inc., San Jose, CA). Mean values of data underwent t-test or one-way analysis of variance (ANOVA) repeated measures and were compared by the all pairwise multiple comparison Holm–Sidak test; *p* < 0.05 values were considered statistically significant.

The fitting of the excitation fluorescence spectra of SCL and SCT standard compounds at pH 5.4 was performed by using a single-Gaussian curve with a 3-parameter function:


(1) $$f=a\,e^{-\left[\frac{1}{2}\cdot {\left(\frac{x-x_{0}}{b}\right)}^{2}\right]}$$

where, *a* is the amplitude of the curve band, *x*_0_ is the central wavelength, and *b* is the standard deviation of a normal data distribution, which is related to the full width at half maximum (FWHM) of the band by *b* ≈ 2.355/FWHM. In the fitting, these three parameters were set to be free, without any constraint.

For the SCT coumarin at pH 8.5, the linear combination of two-Gaussian curves with a user-defined function:


(2) $$f=a\,e^{-\left[\frac{1}{2}\cdot {\left(\frac{x-x_{0}}{b}\right)}^{2}\right]}+c\,e^{-\left[\frac{1}{2}\cdot {\left(\frac{x-x_{1}}{d}\right)}^{2}\right]}$$

was applied. So that three additional parameters *c*, *x*_1_, and *d*, corresponding to the amplitude, central wavelength, and standard deviation of the second Gaussian curve were added. In the fitting, the central wavelength and bandwidth of one curve, *x*_0_ and *b*, respectively, were set close to those found for SCT at pH 5.4, while the other four parameters were set to be free.

The *in vivo* fluorescence excitation spectra measured on leaf disks were fitted with the two-Gaussian curve (Eq. 2).

The coefficient of determination, *R*^2^, was used to estimate the fitting quality.

## Results

**FIGURE 1:**
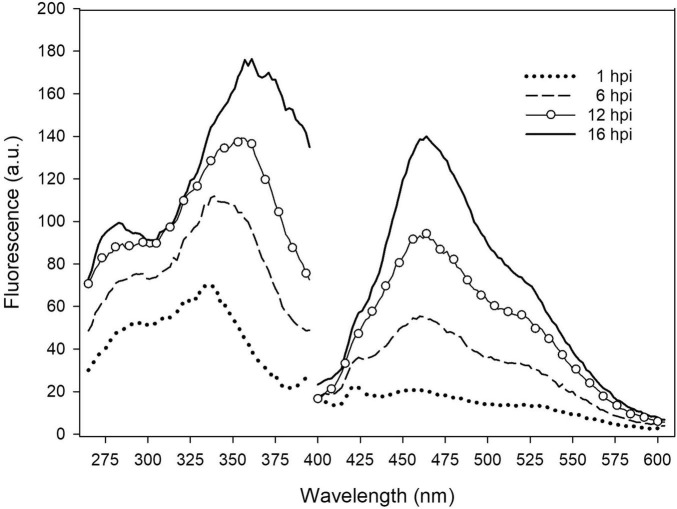
*In situ* emission and excitation fluorescence spectra of tobacco leaf disk infiltrated with *P. syringae* pv. *tomato* DC3000 recorded on the adaxial side at different hpi. The emission spectra were acquired under excitation at 370 nm. The excitation spectra were acquired with emission at 460 nm. Each spectrum is the average of 6 measurements; a.u., arbitrary units.

The early responses of the tobacco leaves during the interaction with several *P. syringae* strains was evaluated by measuring *in situ* the excitation and emission fluorescence spectra of leaf disks. In Figure 1, the spectra acquired on samples at different times (h) post-infiltration (hpi) with *P. syringae* pv. *tomato* DC3000 are reported.

The leaf fluorescence emission and excitation spectra were both observed to significantly increase with post-infiltration time. Interestingly, while the shape of the emission spectra, peaked at 460 nm, did not change with time, the main band of the excitation spectra shifted from around 335 nm to 360 nm, receiving a larger contribution at longer wavelengths.

### Confocal Microscopy

**FIGURE 2:**
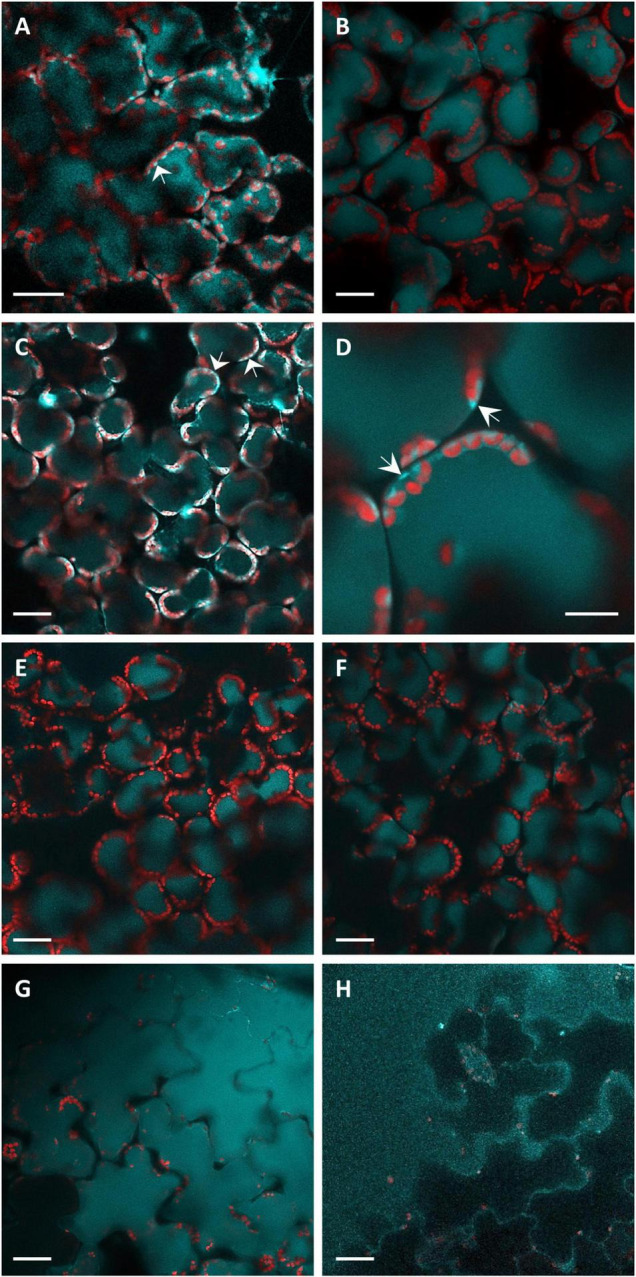
The CLSM fluorescence imaging of leaf disks observed from the adaxial side. Light-blue fluorescence, acquired under excitation at 405 nm and detection at 430–498 nm, represents localization of coumarins. Red fluorescence coming from the chlorophyll of the chloroplasts was acquired under excitation at 638 nm and detection at 695–765 nm. Images were acquired at 9 **(A)**, 22 **(B)**, 28 **(C,G)**, 31 **(E,H)**, 48 **(D)**, and 51 **(F)** hpi. **(A–D,G)** Report images of *P. syringae* pv. *tomato* DC3000 infiltrated leaf disks. **(E,F,H)** Report the images of SPS-infiltrated leaf disks (controls). The bar in **(D)** represents 10 μm, all the other panel bars represent 30 μm. The arrows indicate the cytoplasmatic localization of blue fluorescence.

Knowing the distribution of fluorophores inside the leaf tissues is fundamental for the analysis of the *in situ* fluorescence spectra. Therefore, we performed confocal microscopy analyses of leaf disks infiltrated by *P. syringae* pv. *tomato* DC3000 or SPS as shown in Figure 2. The intracellular localization of blue autofluorescence, under excitation at 405 nm, appeared rather heterogenous between vacuole and cytoplasm depending on the treatment and the time post-inoculation. Some samples presented strictly vacuolar localization of blue fluorescence (Figures 2B,E,F). Others clearly showed the presence of intense cytoplasmatic blue fluorescence, besides vacuolar fluorescence, as “lenticels” shape structures confined near to plasma membrane (Figures 2A,C,D). In the bacterial infiltrated samples, a much higher fluorescence than that of the SPS-infiltrated samples was observed in the epidermal cells (Figure 2G vs. Figure 2H). The control SPS-infiltrated leaf disks showed fluorescence only in the vacuole, even long time (i.e., 51 hpi) after the treatment (Figures 2E,F).

The leaf fluorescence assignment to specific compounds during the interaction with pathogens can be better addressed on the basis of the *N. tabacum* leaf phytochemical composition and the *in vitro* absorption and fluorescence properties of the major constituents.

### Main Phenolic Compounds in Untreated and Infiltrated Leaves

**FIGURE 3:**
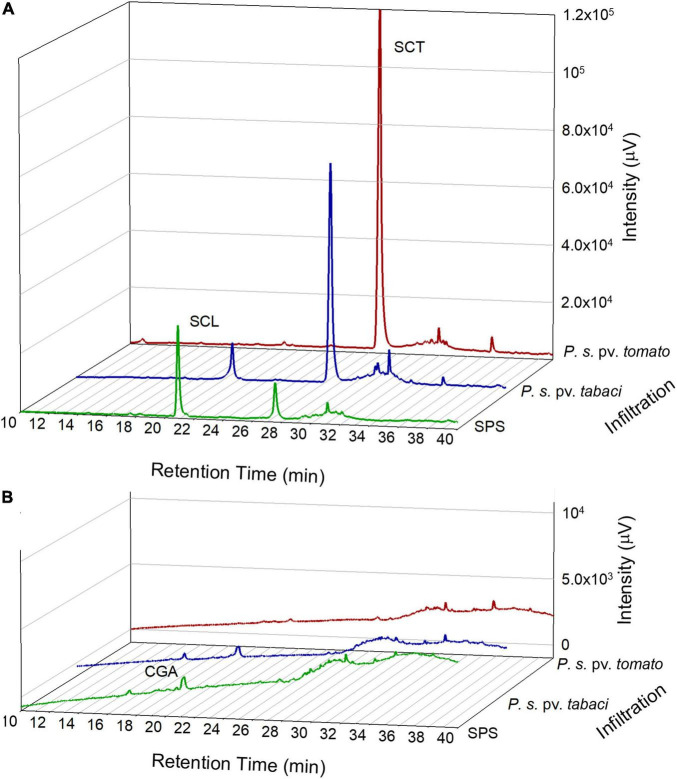
Representative chromatograms from the HPLC analysis of tobacco leaf disk extracts acquired by a fluorescence detector, excitation at 340 nm and emission at 460 nm **(A)** and by a diode array detector at 330 nm **(B)**. Leaf samples were analyzed at 18 hpi with SPS, *P. syringae* pv. *tabaci* ATCC 11528 or *P. syringae* pv. *tomato* DC3000.

The polyphenolic composition of tobacco leaves was determined by the HPLC analysis of leaf disk extracts collected before and after the bacterial infiltration. In Figure 3, chromatograms representative of the leaf samples at 18 hpi are reported. The principal phenolic compounds were CGA, SCL, and SCT. Some other minor peaks appeared at retention times higher than 30 min. These compounds could not be definitely identified because of their low contributions to the HPLC absorbance signals that impaired the acquisition of proper DAD spectra. Their detection by tandem mass spectrometry did not provide conclusive results. We estimated, however, that in the fluorescence chromatograms the sum of the unidentified peaks relative to those of SCL plus SCT was less than 10% on average. Therefore, we consider them not relevant.

The HPLC–MS/MS analysis confirmed the presence of CGA, SCL and SCT according to their fragmentation pattern as reported in Supplementary Table 1.

**FIGURE 4:**
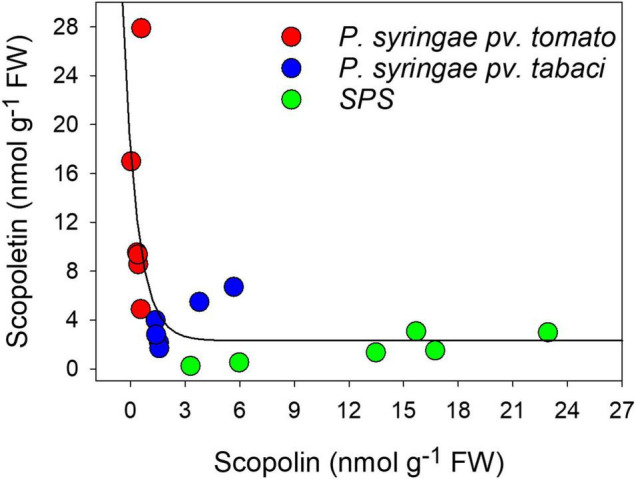
Relationship between SCT and SCL concentrations and the infiltration treatments, evaluated by chromatographic methods. The solid line represents a curve fitting by an exponential decay function.

By plotting the concentrations of the two coumarins against each other, we found that they were related by an exponential decay function (Figure 4) indicating that the maximal level of SCT corresponded to the minimum level of SCL.

Figure 4 also shows the content of the coumarins as function of the infiltration treatment. When leaf disks were only infiltrated with SPS, the highest levels of SCL and the lowest levels of SCT were found, likely due to the stress induced by cutting. Conversely, the largest and the lowest amounts of SCT and SCL, respectively, were observed after infiltration with *P. syringae* pv. *tomato* DC3000, which is avirulent on tobacco. At last, intermediate values of both these two coumarins were obtained after leaf infiltration with *P. syringae* pv. *tabaci* ATCC 11528, the virulent bacterium for tobacco.

### *In vitro* Fluorescence of Standard Compounds

To identify the principal contributors to the tobacco leaf fluorescence under pathogen infiltration, we analyzed the fluorescence properties of the main phenolic compounds found by the HPLC analysis.

**FIGURE 5:**
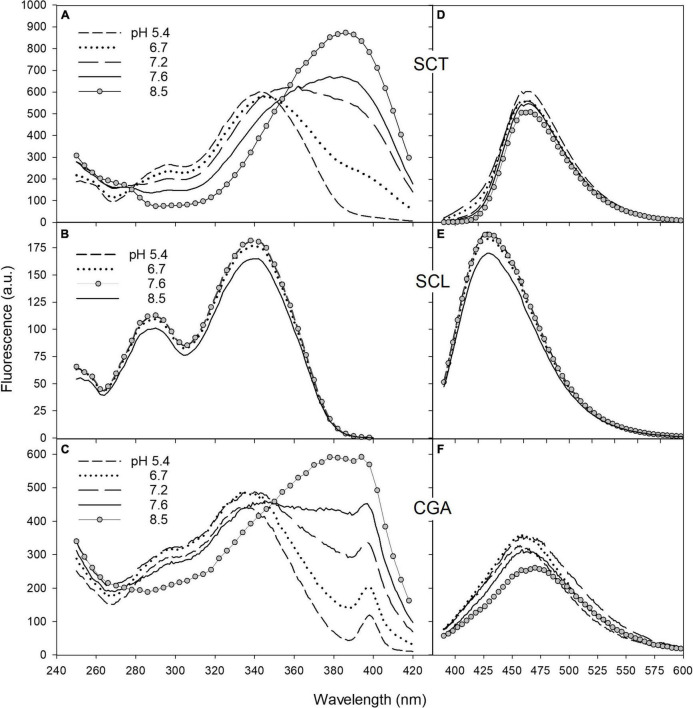
The fluorescence excitation and emission spectra of SCT **(A,D)**, SCL **(B,E)**, and CGA **(C,F)** in phosphate buffer at different pH. Excitation spectra were recorded with emission wavebands set at 460 nm for SCT and CGA and at 420 nm for SCL. The peak observed in C at 398 nm corresponds to the excitation wavelength of the Raman scatter band of water at 460 nm. For emission spectra, excitation wavelengths were set at 330, 340, and 350 nm for CGA, SCL, and SCT, respectively. The acquisition parameters for SCT and SCL were ex/em slit width of 2.5/5 nm for excitation, 5/2.5 nm for emission, PMT voltage = 600 V; for CGA, they were ex/em slit width of 5/10 nm for excitation, 10/5 nm for emission, PMT voltage = 900 V.

Since coumarin fluorescence is strongly pH-dependent (Fink and Koehler, 1970; Pina et al., 2019; Pham et al., 2020), we investigated the fluorescence properties of SCT and SCL, and also CGA, in buffer solutions of different acidity. The excitation and emission spectra of SCT, SCL, and CGA in phosphate buffer at various pH values are reported in Figure 5. Moving the pH from 5.4 to 8.5, the excitation spectrum of SCT showed a decrease of the peak around 340 nm and a relative increase of the band at 385 nm (Figure 5A). The same pH-dependence was observed for the SCT absorption spectra (see Supplementary Figure 1).

The emission band peaked at 460 nm and remained unchanged with increasing pH (Figure 5D).

Scopolin was characterized by a fluorescence excitation spectrum with a main peak at around 340 nm and a second band at 290 nm (Figure 5B). This spectrum matched closely the absorption spectrum and was not affected by pH change.

The emission band of SCL peaked at 420 nm and was independent of the buffer pH (Figure 5E).

The fluorescence spectra of CGA as function of pH behaved similarly to those of SCT (Figures 5C,F). The intensity of the CGA fluorescence, however, was largely weaker than that of the coumarins. Note that even if in Figure 5, the CGA fluorescence intensity values were similar to those of SCT, the photomultiplier gain and the monochromator slits set in the spectrofluorimeter to measure the CGA signals were much higher than those used to detect the coumarin fluorescence. Nevertheless, the presence of an apparent excitation band at 398 nm corresponding to the Raman scatter band of water at 460 nm in the excitation spectrum of CGA (Figure 5C), not visible in the spectra of SCT and SCL (Figures 5A,B), confirms the weakness of the CGA fluorescence with respect to that of coumarins.

The comparison of the fluorescence signals among the three major phenolic compounds present in the tobacco leaves was further achieved by measuring the relative fluorescence quantum yields. The fluorescence quantum yield of SCT in buffer at pH 5.4 resulted to be about 740 and 3.2 folds higher than that of CGA and SCL, respectively. Scopolin is in turn about 230 folds more fluorescent than CGA. Still, the anionic form of SCT was at least 1.5 times more fluorescent than its neutral form, in accordance with data from the literature (Masilamani and Sivaram, 1981; Pina et al., 2019).

### Gaussian Fitting of Spectra

Since the excitation spectra of SCL and SCT partially overlapped, especially at lower pH values, we performed a Gaussian curve spectral fitting to separate their contribution to the *in vivo* fluorescence.

We considered the spectral range above 300 nm, neglecting the shorter waveband of the excitation spectra (see Figure 5) since *in vivo*, the contribution of UVB-excitation wavelengths to the blue fluorescence of leaf cells is expected to be minimal due to the large absorption in this region by the cuticle, waxes, and trichomes of the outer layers of the leaf.

**FIGURE 6:**
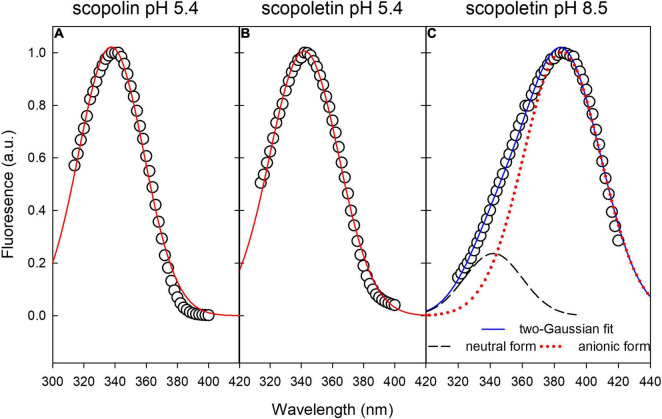
Gaussian fitting of excitation fluorescence spectra of standard compounds in buffer solution. **(A,B)** Single-Gaussian curve fitting of SCL and SCT at pH 5.4, respectively. **(C)** Two-Gaussian curve fitting to take into account the residual component from the neutral form of SCT.

Excitation fluorescence spectra of SCL and SCT standard compounds at pH 5.4 can be fitted by a single-Gaussian curve (Figures 6A,B). The excitation spectrum of SCT at pH 8.5 was fitted by the sum of two-Gaussian functions (Figure 6C) to take into account the residual contribution from the neutral molecular species of the acid-base equilibrium present in solution (Pina et al., 2019). The FWHM of all the Gaussian curves was around 52 nm, while the peak wavelength was 338, 342, and 385 nm for SCL at pH 5.4, SCT at pH 5.4, and SCT at pH 8.5, respectively. The coefficient of determination, R^2^, of the fitting varied between 0.994 and 0.996.

Based on this, we were able to perform the spectral fitting of *in vivo* leaf fluorescence excitation spectra by two-Gaussian curves centered at around 360 and 385 nm.

Before leaf disk punching and infiltration, the excitation spectra were recorded *in planta*, on the very same leaves, then detached and used for the processing. These spectra were subtracted from those measured on the leaf disks at different times after infiltration to take scattering effects due to the leaf structures into account.

**FIGURE 7:**
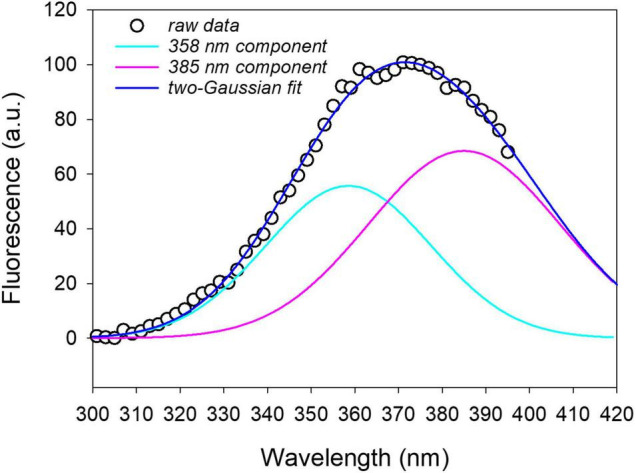
Two-Gaussian curves fitting of the fluorescence excitation spectrum (emission at 460 nm) measured on a leaf disk after 16 h from infiltration with *P. syringae* pv. *tomato* DC3000. The on plant average excitation spectrum was removed from the leaf disk spectrum before fitting. *R*^2^ = 0.997.

An example of the spectral fitting is reported in Figure 7 for the excitation fluorescence spectrum recorded on a leaf disk at 16 hpi with *P. syringae* pv. t*omato* DC3000.

### Correlation Between Fluorescence Signals and the Leaf Coumarin Content

The two-Gaussian fitting of excitation spectra (for emission at 460 nm) was applied to the data measured on the different leaf disk samples that were then processed for the HPLC analysis of phenolic compounds. The samples selected were those infiltrated with *P. syringae* pv. *tomato* DC3000, *P. syringae* pv. *tabaci* ATCC11528, or SPS, both at 18 and 24 hpi, derived from three different leaves of three different tobacco plants.

**FIGURE 8:**
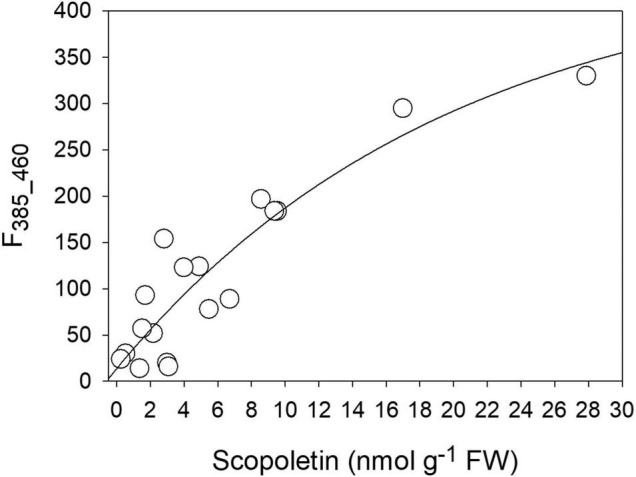
The correlation between the intensity of the 385 nm fluorescence excitation band (F_385_460_) and the SCT concentration as evaluated by chromatographic methods.

By comparing the concentration values of the different compounds obtained by the HPLC analysis with the intensity of the two fluorescence bands derived by the Gaussian fitting of the spectra, a good correlation was found between SCT and the band at 385 nm (F_385_460_). The direct relationship could be fitted by an exponential function [*F*_385_460_ = 13.81 + 436.9 × (1 - *e*^–0.05C^), with C = SCT concentration] with *R*^2^ = 0.84 (Figure 8). Both SCL and CGA did not show any direct correlation to either the 360 nm or the 385 nm fluorescence excitation bands (see Supplementary Figure 2).

### Time-Course Monitoring of the Bacteria–Plant Interaction

**FIGURE 9:**
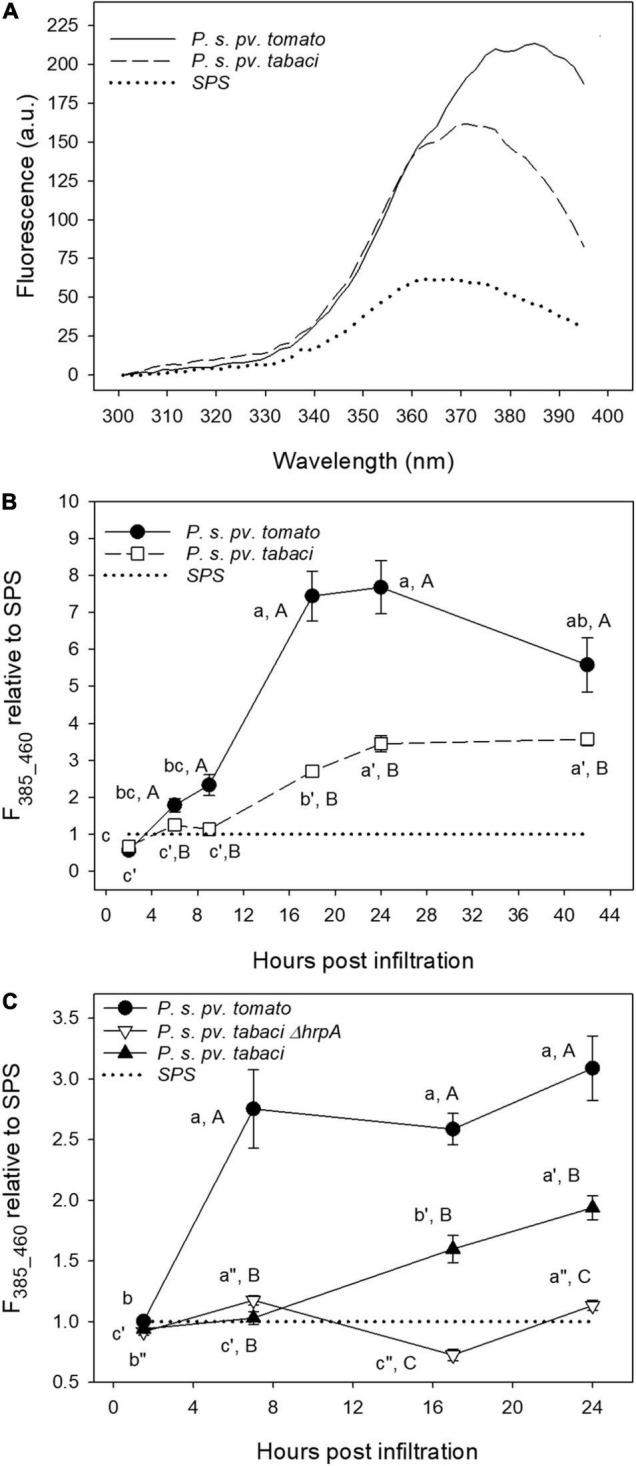
The fluorescence excitation spectra of tobacco leaf disks measured at 24 hpi **(A)**, and time evolution of the 385 nm fluorescence excitation band (emission at 460 nm, F_385_460_) **(B,C)**. The leaf disks were separately infiltrated with *P. syringae* pv. *tabaci* ATCC11528, its *ΔhrpA* mutant, *P. syringae* pv. *tomato*, and with SPS as negative control. Each point is the mean (±SE) of leaf disks (*n* = 24–45) from three different leaves of three different plants. The average excitation spectrum *in planta* on attached leaves was subtracted from the leaf disks spectra. In **(B,C)**, the values marked by a different lower case letter and primed or double-primed letter for the same treatment, and by an upper case letter for each time are significantly different (*p* < 0.05) according to the Holm–Sidak test.

We applied the deconvolution of the excitation spectra by two-Gaussian fitting to the spectra measured on leaf disks under different bacterial treatments at increasing post-infiltration time. In Figure 9A, the average excitation fluorescence spectra, for emission at 460 nm, of leaf disks infiltrated with *P. syringae* pv. *tomato* DC3000, *P. syringae* pv. *tabaci* and SPS at 24 hpi are shown.

Figure 9B displays the time-course of the F_385_460_ fluorescence excitation band, determined by the previously described fitting spectral analysis, representing the evolution of SCT, and normalized to that of control samples.

Additionally, we compared the F_385_460_ fluorescence excitation band induced by the previous treatments with that generated by the *ΔhrpA* mutant of *P. syringae* pv. *tabaci* ATCC11528 (Figure 9C).

The *P. syringae* pv. *tomato* DC3000 treatment induced a faster and much higher increase of the fluorescence intensity with respect to that caused by *P. syringae* pv. *tabaci* ATCC11528 infiltration. A plateau is reached even as early as 7 hpi (Figure 9C), and a decreasing trend was observed after 24 hpi (Figure 9B). Then F_385_460_ relative to SPS in leaf disks infiltrated with *P. syringae* pv. *tabaci* ATCC11528 increased linearly from 7 to 9 hpi up to 24 hpi and then remained stable. Under the infiltration with its *ΔhrpA* mutant, *F*_385_460_ oscillated with time remaining close to that of the SPS control (Figure 9C). At 17 and 24 hpi, it was significantly lower than the fluorescence induced by the *P. syringae* pv. *tabaci* ATCC11528 wild type strain.

## Discussion

The *in situ* tobacco leaf fluorescence spectroscopy during the first hours of the plant–bacteria interaction showed clearly that the excitation spectra changed shape with time (Figure 1).

The possible contributors to the observed leaf blue fluorescence were identified to be SCL and SCT, as resulted by the HPLC analysis of leaf disk extracts collected before and after the bacterial infiltration, as well as by the *in vitro* fluorescence properties of these compounds.

The levels of SCL and SCT in untreated tobacco leaves were low, ranging 70–154 ng g^–1^ FW and 15–38 ng g^–1^ FW, respectively. The concentration of CGA ranged 330–600 μg g^–1^ FW. This was consistent with the CGA levels previously found in *N. tabacum* leaves (Baker et al., 2017), considering also its high variability due to the growing conditions, leaf age and position on the plant.

After bacterial infiltration, the concentration of both SCL and SCT significantly increased up to 0.56 and 5.36 μg g^–1^ FW, respectively, while that of CGA markedly decreased. In SPS-infiltrated leaves, SCL reached the maximal concentration of 8.13 μg g^–1^ FW, while SCT was utmost 0.59 μg g^–1^ FW. A high variability between plants in the coumarin accumulation was, however, observed as function of the type of bacteria, the time after bacterial infiltration and the mock infiltration with SPS.

### Fluorescence Properties of Tobacco Phenolic Compounds

The SCT excitation spectra showed the typical changes with pH observed for the acid–base equilibrium of mono-hydroxycoumarins (Simkovitch et al., 2016). Increasing the pH from 5.4 to 8.5, the neutral form of SCT, with excitation band at around 340 nm, decreased while a relative increase of the anion band at longer wavelengths, at 385 nm, appeared (Figure 5A). The excitation spectrum of SCT at pH 8.5 can then be considered as due to the pure anionic molecular species, in accordance with the literature (Pina et al., 2019; Pham et al., 2020).

The emission band peaking at 460 nm remained unchanged with increasing pH (Figure 5D) because, in the considered pH range, the intensity of the neutral form near 430 nm is much weaker than that of the anion. In fact, SCT is defined as a photoacid, which means that its excited neutral form can easily lose a proton and fluoresce as the anion. Accordingly, the ground-state pKa of SCT of 7.4 is reduced to about 1 in the excited-state (Pina et al., 2019; Pham et al., 2020). This process can occur through the intermolecular excited-state proton-transfer (ESPT) to water.

Scopolin was characterized by the fluorescence excitation and emission spectra independent of pH (Figures 5B,E) due to the glucoside-substitution of the hydroxy group in position 7 that does not allow any deprotonation of the molecule.

The pH-dependence of the CGA fluorescence spectra (Figures 5C,F) was similar to that of SCT. This indicates that CGA can be considered a photoacid and undergoes to ESPT as observed in other phenolic compounds (Kaneko et al., 2009).

The *in vitro* fluorescence analysis, both considering spectral shape and relative quantum yields of compounds, suggested SCL and SCT as the main contributors to the leaf blue fluorescence observed under bacterial infiltration. Yet, the excitation spectrum for emission at 460 nm seems to be the most useful tool to discriminate the two coumarins during the plant–pathogen interaction, based on its specific responsiveness to pH changes. These results further support the hypothesis that the leaf blue fluorescence of bacterial inoculated samples receive contributions from the vacuolar SCL and the extra-vacuolar SCT, as evidenced by confocal microscopy (Figure 2) and discussed in the following section.

### Leaf Tissue Localization and Diffusion of Coumarins

The confocal microscopy analysis of leaf disks infiltrated by *P. syringae* pv. *tomato* or SPS showed the localization of blue autofluorescence in the vacuole and cytoplasm of palisade cells, as well as in the adaxial epidermal cells, depending on treatment and time after infiltration. Therefore, the fluorophores at all these leaf tissue compartments can contribute to the in situ measured leaf fluorescence.

Microimages of vacuolar blue fluorescence, excited at 364 nm, similar to those we found were observed and assigned to SCL in the epidermal and mesophyll cells of leaves from *Arabidopsis thaliana* plants grown for 4 weeks at 20°C and then kept for 1 week at 10°C (Döll et al., 2018).

It is widely recognized that the toxic SCT is converted by glucosyltransferases to the glycosylated form SCL (Ibrahim and Boulay, 1980), which is transported to the vacuoles for storage. In response to the defense activation induced by a pathogen or elicitor, SCL can be excreted into the cytosol and apoplast and converted back to SCT by β-glucosidases (Stringlis et al., 2019). In this way, SCT can exert its antimicrobial activity and scavenge H_2_O_2_ in the infected tissues (Chong et al., 1999).

On the other hand, SCL possesses lower antioxidant activity than SCT (Al-Rifai, 2018) because of the glucosylation of the reactive hydroxyl group at position 7. Also, SCL showed less-effective inhibitory capacity than its aglycone toward various fungal and bacterial pathogens (Ahl Goy et al., 1993; Beyer et al., 2019). Therefore, it is expected that the tobacco plant response to biotic oxidative stresses will favor the accumulation of SCT rather than SCL.

In *Hevea brasiliensis*, leaves inoculated with *Phytophthora palmivora* excreted a single coumarin compound, identified as SCT, in the droplets recovered from the inoculated site (Churngchow and Rattarasarn, 2001).

Taguchi et al. (2000) proved the uptake of SCT added to the culture medium of tobacco cells treated with the synthetic auxin 2,4-dichlorophenoxyacetic acid. Scopoletin was mostly completely glucosylated to SCL in the cytoplasm, and transferred to the vacuole. Only a 2% fraction of SCT with respect to SCL was determined in the cell vacuoles.

The process of conversion from SCL to SCT and their different compartmentalization in response to biotic stresses to scavenge reactive oxygen species (Chong et al., 1999; Stringlis et al., 2019) is also supported by the exponential decay relationship we found between SCT and SCL concentrations (Figure 4), indicating that the maximal level of SCT corresponded to the minimum level of SCL.

Based on these results, we hypothesize that the intense blue autofluorescence observed in the cytoplasm of our samples was due to the presence of SCT produced after excretion of SCL from the vacuole following the infiltration with *P. syringae* pv. *tomato*. The vacuolar fluorescence instead was mainly due to SCL. This is consistent with the *in vitro* fluorescence properties of coumarins (Figure 5) considering that while the vacuolar pH in higher plant cells is typically between 5.0 and 6.0, higher pH values up to 7.5 could be measured in the cytoplasm (Kurkdjian and Guern, 1989; Martinière et al., 2013).

The increase of fluorescence in the epidermal cells after bacterial infiltration can be explained by the transportation of defense compounds from the synthesis site through symplasmic domains toward the leaf edges (Jørgensen et al., 2015). Accordingly, both SCL and SCT (and also ayapin) were found on the surface of fungi-infected sunflower leaves (Prats et al., 2007). The resistance to rust infection induced in sunflower by acibenzolar-S-methyl was correlated to an increased synthesis of coumarins and their excretion to the leaf surface, especially in the case of SCT (Prats et al., 2002). Other hydroxylated coumarins, such as esculin and esculetin, were found in the vacuole of subsidiary cells of stomata and guard cells of the epidermis and the mesophyll protoplasts in *Arabidopsis thaliana* (Rottmann et al., 2018) and in the vacuole of epidermal and palisade cells of *Fraxinus ornus* leaves (Tattini et al., 2014).

The dynamics of compound diffusion among cell compartments during the plant–bacteria interaction (Baker et al., 2018) or in tobacco leaf tissues treated by HR-inducing elicitors (Dorey et al., 1997) can explain the heterogenous response in term of the tobacco leaf phenolic composition during the bacterial and mock treatments (the range of concentrations found at 18 and 24 hpi was 0.26–99.67, 0.15–81.88, and 0.24–27.87 nmol g^–1^ FW for CGA, SCL, and SCT, respectively).

### The Scopoletin F_385_460_ Fluorescence as Marker of the Plant–Bacteria Interaction

The two-Gaussian fitting deconvolution of fluorescence excitation spectra was able to discriminate the contribution of SCT from that of SCL to total leaf fluorescence. This was proved by the good direct correlation found between the F_385_460_ signal and the concentration of SCT determined destructively (Figure 8). On the other hand, the other main phenolic fluorophores present, SCL and CGA, did not show any direct correlation with F_385_460_ (Supplementary Figure 2). We could then use the developed methodology to monitor *in vivo* the evolution of SCT during a 42-h period of the plant–pathogen interaction (Figures 9B,C). A large difference in the leaf blue autofluorescence intensity and spectral shape between the various trials was found. This was likely due to differences in leaf and plant age, as well as in the general plant physiological state. In fact, the production of SCT was proved to increase in tobacco resistant cultivars after infection with *P. syringae* pv *tabaci* depending on an increase in cytokinins concentration (Großkinsky et al., 2011). Since the content of cytokinins is markedly dependent on the leaf position on the plant, decreasing from the uppermost leaves to the bottom (Veselov et al., 1999), it is expected to find rather quantitative different responses to the pathogen infiltration as a function of the leaf and plant age.

The common aspects observed among the trials concerned two main differences related to the resistance response of tobacco to avirulent bacterial Gram negative plant pathogens. The shape of the excitation spectrum at 24 hpi peaked at around 380–390 nm only on samples infiltrated with *P. syringae* pv. *tomato* DC3000. Moreover, a clear and faster accumulation of extra-vacuolar SCT was detectable under *P. syringae* pv. *tomato* DC3000 than under *P. syringae* pv. *tabaci* infiltration.

Overall, our results clearly show that the amount and the timing of SCT accumulation, estimated by F_385_460_, is linked to the level of tobacco resistance to the tested pathogens.

The rapid rise of the SCT concentration following *P. syringae* pv. *tomato* DC3000 infiltration is consistent with early studies on the accumulation of SCT in attached tobacco leaves treated with an elicitor of the hypersensitive reaction (Dorey et al., 1997). They reported an increase of this coumarin in the tissue surrounding the infiltration zone already 6 h after the treatment, reaching a maximum after 24 h. In the elicitor-treated zone, SCT slightly increased because of its high sensitivity to oxidation. The delayed increase of the F_385_460_ fluorescence in tobacco leaf disks infiltrated with *P. syringae* pv. *tabaci* ATCC11528 with respect to *P. syringae* pv. *tomato* DC3000 (Figures 9B,C) is a typical feature of the defense responses triggered into a plant host against a virulent pathogen, allowing the infection to progress. The plant barriers against an avirulent pathogen, such as *P. syringae* pv. *tomato* DC3000 with tobacco, are rapidly activated and their effects are evident from the first few hours of post-infiltration. It is worth to mention that a functional TTSS is essential to trigger accumulation of coumarins in tobacco tissues by a Gram negative plant pathogenic bacterium. No significant increase of F_385_460_ (i.e., SCT) was ever observed in tobacco leaf disks infiltrated with the non-pathogenic *ΔhrpA* mutant of *P. syringae* pv. *tabaci* ATCC11528. This effect is in accordance with the additional role of the HrpA protein. Besides to be the major component of the TTSS pilus, HrpA has been demonstrated to positively affect TTSS gene expression in phytopathogenic bacteria belonging to the *P. syringae* complex, by acting upstream to the expression cascade of TTSS genes, that is on the hrpRS operon (Wei et al., 2000; Tang et al., 2006; Ortiz-Martín et al., 2010; Tegli et al., 2011). Therefore, these overall data are further confirming both sensitivity and reliability of the fluorimetric method developed in this study.

## Conclusion

We developed a new fluorescence spectroscopic method able to selectively control *in vivo* the evolution of the SCT phytoalexin following a bacterial infiltration of tobacco leaves. It is based on the elaboration of the fluorescence excitation spectra recorded at different times during the elicitation process. The key point of the method relies on the dependence of the SCT fluorescence excitation spectrum on pH. This allowed the detection of an excitation fluorescence band around 385 nm specific of SCT located in the cytoplasmatic cell compartment and free of the SCL contribution.

The Gaussian curve deconvolution analysis of leaf blue autofluorescence excitation spectra permitted, for the first time, separating the contribution to leaf fluorescence of SCL from that of SCT. It represents a fundamental improvement in the development of non-destructive methods for the early diagnosis of tobacco plant diseases and in the discrimination of the plant response to specific pathogens and likely to other stresses.

The outcome of the present study can be exploited to define a screening technique for the early diagnosis of plant diseases, even in the field by using suitable portable fluorescence sensors. It could also find application for an early and rapid selection of tobacco cultivars resistant against virulent pathogens.

## Data Availability Statement

The raw data supporting the conclusions of this article will be made available by the authors, without undue reservation.

## Author Contributions

GA and ST: conceptualization, funding acquisition, and project administration. GA: data curation, formal analysis, software, and writing the original draft. CB, ID, and ST: investigation, validation, and reviewing and editing the content. LT: investigation. All authors contributed to the article and approved the submitted version.

## Conflict of Interest

The authors declare that the research was conducted in the absence of any commercial or financial relationships that could be construed as a potential conflict of interest.

## Publisher’s Note

All claims expressed in this article are solely those of the authors and do not necessarily represent those of their affiliated organizations, or those of the publisher, the editors and the reviewers. Any product that may be evaluated in this article, or claim that may be made by its manufacturer, is not guaranteed or endorsed by the publisher.
