# Supplementary material for: Retrieving the in vivo Scopoletin Fluorescence Excitation Band Allows the Non-invasive Investigation of the Plant–Pathogen Early Events in Tobacco Leaves
Source: Front Microbiol. 2022 Apr 29;13:889878. doi: 10.3389/fmicb.2022.889878 (PMC9100583; doi:10.3389/fmicb.2022.889878)
Supplement: Supplementary file 1 [file Table_1.DOCX]

Table S1. Results of the HPLC-ES-MS/MS analysis of leaf extracts

| **Compound name** | **Retention time (min)** | **Raw formula** | **Molecular ions (the precursor ion for MS2 is in bold)** | **Product ions (the highest product ions are in bold)** |
| --- | --- | --- | --- | --- |
| Chlorogenic acid  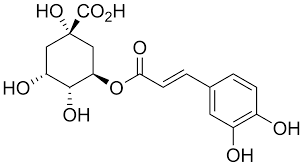 | 7.7 | C_16_H_18_O_9_ | **353.0870** **[M-H]^-^** | **191.0543** |
| Scopolin  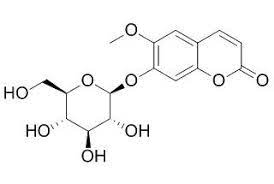 | 9.4 | C_16_H_18_O_9_ | **355.102 [M+H]^+^**  and 377.084 [M+Na]^+^ | **193.049**, 178.025, 166.057, 133.027 |
| Scopoletin  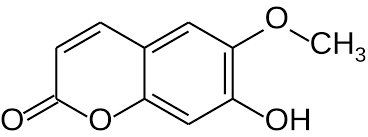 | 12.0 | C_10_H_8_O_4_ | **193.049 [M+H]^+^**  and 215.031 [M+Na]^+^ | 178.025, 150.030, 133.028, **122.0354** |
