# Supplementary material for: Retrieving the in vivo Scopoletin Fluorescence Excitation Band Allows the Non-invasive Investigation of the Plant–Pathogen Early Events in Tobacco Leaves
Source: Front Microbiol. 2022 Apr 29;13:889878. doi: 10.3389/fmicb.2022.889878 (PMC9100583; doi:10.3389/fmicb.2022.889878)
Supplement: Supplementary file 2 [file Presentation_1.PPTX]

## Slide 1
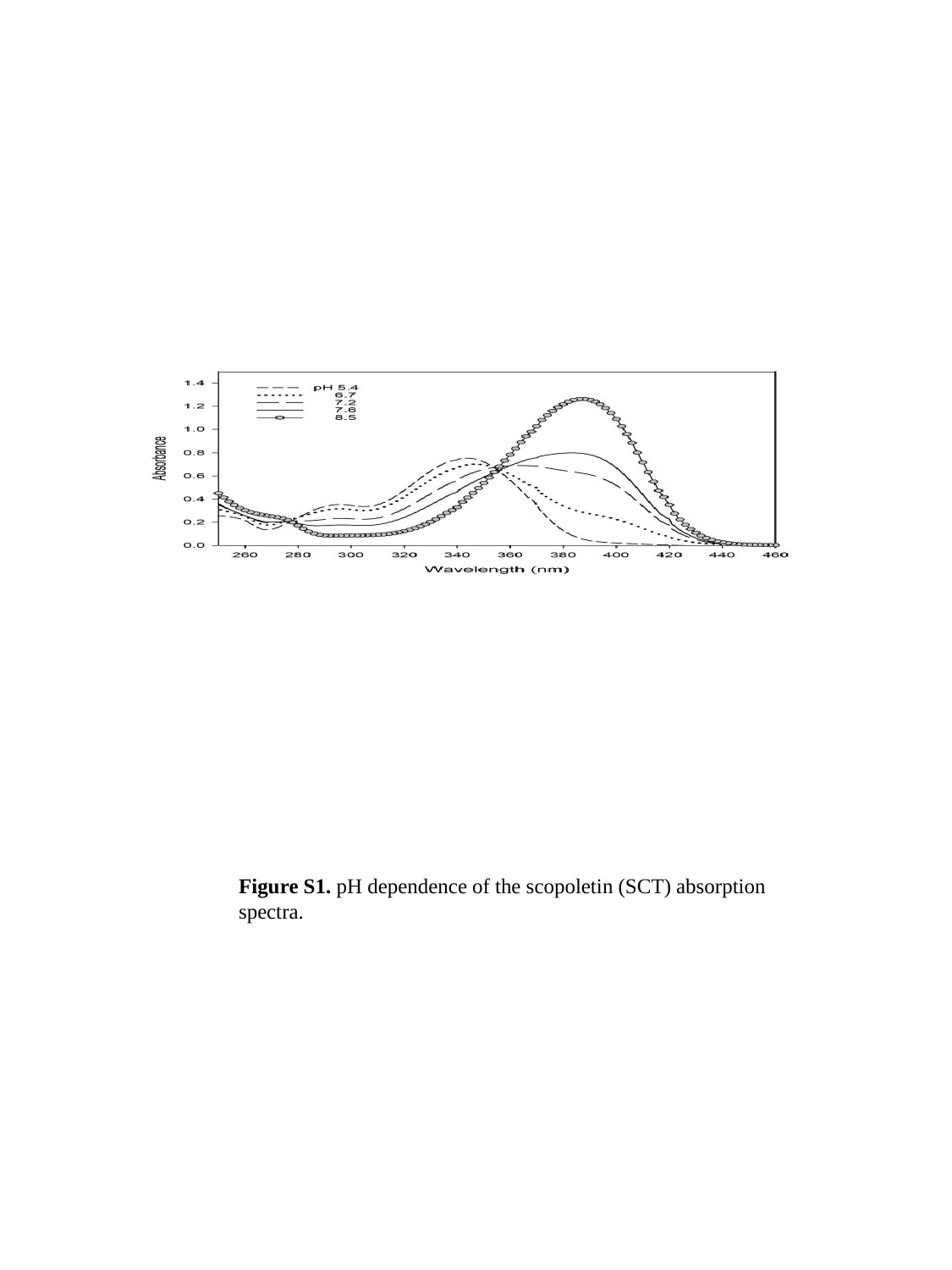

Figure S1. pH dependence of the scopoletin (SCT) absorption spectra.

## Slide 2
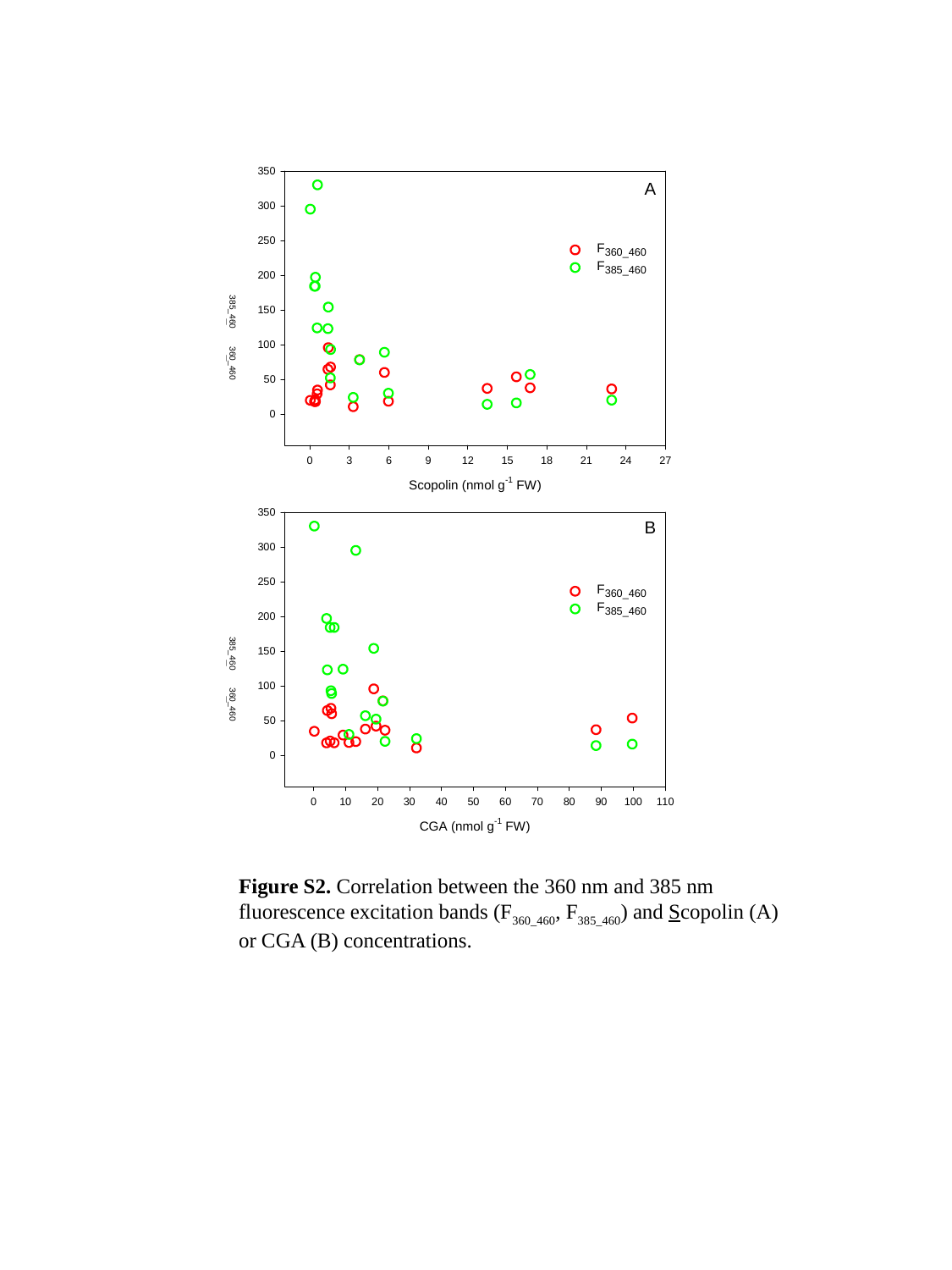

Figure S2. Correlation between the 360 nm and 385 nm fluorescence excitation bands (F360_460, F385_460) and Scopolin (A) or CGA (B) concentrations.
